# Tuberculous Disease of the Knee-Joint

**Published:** 1908-10-03

**Authors:** 


### Surgery.

### TUBERCULOUS DISEASE OF THE KNEE-JOINT.

A tuberculous knee is an affliction commonly associated with childhood or with early adult life; but no age can be called exempt. This is characteristic of tuberculosis, especially as it attacks bones or joints. To take another example, it is a matter of common knowledge that caries of the spine is stated to be a disease of the earlier decades of life; and yet one is constantly surprised to come across cases in which this disease has made its appearance in patients over the age of forty, in whom there was no previous indication of its presence.

The disease starts in the knee, as in other joints, in one of three places—(*a*) in the synovial membrane itself, (*b*) in the bone, on the diaphysial side of the epiphysial line, or (*c*) just under the cartilage which covers the articular surface. As far as we know, the second of these is most common; though such a statement must necessarily be guess-work, because we have such few opportunities of examining the affected joints in a very early stage. The statement, however, is generally accepted as true, and is explained on the ground that in this situation there is a fine network of capillary vessels in the terminal branches of which the bacilli floating in the blood-stream are trapped and form, so to speak, a bacillary embolus.

For it must be supposed that each and all of us have in our circulatory system a number of hostile micro-organisms, which are inert as long as our resistance to their activity is maintained at a normal standard. But if from any cause that resistance becomes lowered, the micro-organisms are at once able to exert their malign influence. This is one of the essential secrets of all pathology; and examples of it could be multiplied a hundredfold. Everyone, for instance, carries in his pharynx the diplococcus of lobar pneumonia; but it is only when his resistance is lowered, as, for the sake of example, by a chill, or, to use a layman's phrase, when he is "run down," that he exhibits the clinical phenomena of the disease. In the same way, in tuberculosis of the knee the diminution of the patient's resistance locally is nearly always the result of the same thing—namely, an antecedent injury. In nearly every case a history of some injury can be obtained; sometimes this occurred only a short time ago, but in others at quite a distant date; and here is a curious fact, that the injury is more often of a slight than of a severe nature. This may seem at first sight a paradox, but the explanation is not far to seek. A severe injury is followed by inflammatory changes, which induce primarily an increased flow of blood to the part, and this blood has bactericidal properties; but a slighter damage merely diminishes the resistance of the part without producing any compensatory vascular changes.

Clinically the onset of the disease is most insidious. Slight pain or jarring is often the first thing noticed, and this will cause limping, since every step in the act of walking must necessarily communicate a jarring to the limb. Then the movements at the joint become limited and impaired, so that flexion and extension beyond certain angles are associated with pain, and soon the patient will hold the limb in the position of greatest comfort. In the case of the knee this position is one of slight flexion, because in that way the synovial cavity can accommodate more fluid. Later still the patient complains of what are known as "starting pains" at night. Just as he is dozing off to sleep, he is awakened by a sharp and violent pain in the joint. The onset of this symptom is evidence that the articular surfaces are now eroded. What happens is that at the moment of going to sleep the periarticular muscles are relaxed. This allows the eroded cartilages to move, one on the other; pain ensues, and a violent contraction of the muscles is induced in an attempt to keep the eroded surfaces firmly in contact and at rest; but the muscular contraction only succeeds in increasing the pain, and so gives rise to the starting pains. This is not pathognomonic of tuberculosis. It occurs as well in septic joints, especially if the infection is not a very acute one. In fact, in this and in many other of the symptoms described arthritis due to a low staphylococcal infection can and very often does closely simulate tuberculosis.

On inspection a tuberculous knee can be seen to be swollen, as evidenced by the filling in of the normal depressions of the joint, and the swollen joint is smooth and white. The swelling, however, often appears to be greater than it really is because the muscles of the thigh and calf are atrophied, especially if the disease be of some standing. This is partly, but not entirely, due to disuse; but the phenomenon is typical of all joint disease, and especially of tuberculous arthritis. In estimating the amount of swelling it is well, therefore, to take an accurate measurement in all cases, and not to trust to the eye alone.

On palpation the joint is found to have a higher temperature than that on the sound side, and it feels "pulpy." This epithet was commonly used to describe tuberculous swellings in past times, and, if not scientific, it is at least descriptive. It conveys more exactly than any other word can do the sensation given to the hand by such a joint. The synovial membrane is thickened and "boggy." With the exception of the staphylococcal arthritis mentioned above, and, rarely, of gummatous synovitis, it is confined to this disease. The sensation is quite different to that given by any of the conditions in which the joint is distended with fluid, but the synovial membrane itself is not grossly altered as, for instance, in osteoarthritis. For it must be remembered that a considerable effusion of fluid into a tuberculous joint is quite rare, although it sometimes occurs.

Further examination consists in investigating the functions of the joint: thus passive movement is resisted, while a slight jar applied to the foot is extremely painful.

(*To be continued.*)

